# Therapeutic Keratoplasty for *Fusarium* Keratitis

**DOI:** 10.3390/jcm13247775

**Published:** 2024-12-19

**Authors:** David Oliver-Gutierrez, Liliana Gutuleac, Natalia Anglada-Masferrer, Gloria Segura-Duch, Sara Martin, Laia Bisbe, María Teresa Martín-Gómez, Miguel Ángel Zapata, Javier José Puig

**Affiliations:** 1 Ophthalmology Department, Hospital Universitari Vall d’Hebron, 08035 Barcelona, Spain; lilianagutuleac@gmail.com (L.G.); natalia_anglada@hotmail.com (N.A.-M.); sara.martin@vallhebron.cat (S.M.); laia.bisbe@vallhebron.cat (L.B.); miguelangel.zapata@vallhebron.cat (M.Á.Z.); javierjose.puig@vallhebron.cat (J.J.P.); 2 Ophthalmology Department, Innova Ocular Verte Barcelona, 08006 Barcelona, Spain; 3 Ophthalmology Department, Centro de Oftalmología Barraquer, 08021 Barcelona, Spain; glo.segura.duch@gmail.com; 4 Institut Universitari Barraquer, Universitat Autònoma de Barcelona, 08021 Barcelona, Spain; 5 Microbiology Department, Hospital Universitari Vall d’Hebron, 08035 Barcelona, Spain; mariateresa.martin@vallhebron.cat

**Keywords:** fungal keratitis, therapeutic keratoplasty, ocular mycosis, corneal transplantation, *Fusarium*

## Abstract

**Purpose**: This study evaluates the effectiveness of therapeutic keratoplasty for *Fusarium* fungal keratitis and explores the diagnosis and management challenges of this infectious corneal disease. **Methods:** We retrospectively analyzed therapeutic keratoplasty cases at a tertiary hospital for *Fusarium* keratitis when standard treatments failed. **Results**: Five cases of *Fusarium* keratitis, unresponsive to typical antifungal treatments, required keratoplasty due to fast progression and diagnostic difficulties. Post-surgery, all patients had infection resolution without recurrence, but some complications like anterior chamber leakage, graft rejection, and ocular hypertension were managed effectively. Final visual acuity ranged from 0.9 to finger counting. **Conclusions**: *Fusarium* keratitis, often resistant to conventional therapies, may necessitate keratoplasty for resolution. This intervention is crucial for positive outcomes, emphasizing the need for prompt and effective management to prevent severe surgical measures and preserve ocular health.

## 1. Introduction

*Fusarium* keratitis is an aggressive ocular infection that can lead to corneal perforation and even affect the vitreous body [1]. Though relatively uncommon, its incidence is on the rise, especially among contact lens users in developed regions [1,2]. *Fusarium* infections are notorious for their virulence and resistance to treatment, sometimes necessitating therapeutic penetrating keratoplasty (TPK) when medical management fails [3]. This contrasts with bacterial keratitis, which typically responds to pharmaceuticals.

The successful management of *Fusarium* keratitis relies heavily on early suspicion and prompt initiation of antifungal therapies [1,4]. Nevertheless, the insidious nature of the disease and the challenges in timely pathogen identification contribute to progression. This challenge is exacerbated by the notable resistance of *Fusarium* strains to a spectrum of antifungal agents [5]. Consequently, TPK emerges as a pivotal therapeutic strategy, potentially demanding more invasive surgeries, like lensectomy or vitrectomy, in severe cases [1].

TPK, despite its complexities and relatively lower graft survival compared to other transplants [6], remains a critical measure against severe outcomes of *Fusarium* keratitis. This paper presents a case series of five patients illustrating the efficacy of therapeutic keratoplasty when early treatment approaches were not sufficient, demonstrating its importance in preserving vision and preventing irreparable ocular damage.

## 2. Methods

A retrospective review of the surgical database at a tertiary hospital was conducted to identify all cases of therapeutic keratoplasty performed for *Fusarium* keratitis in instances where standard treatments had failed. Once identified, demographic data, photographs, treatment details, causative agents, and visual acuity were retrieved from the electronic medical records. The retrospective review was approved by our center’s ethics committee, and this study adhered to the tenets of the Declaration of Helsinki.

The in vivo confocal microscopy (IVCM) performed in case 5 was performed with Heidelberg Retina Tomograph with Rostock Cornea Module (Heidelberg, Germany).

## 3. Results (Table 1)

### 3.1. Case 1 (Figure 1A–C)

**Figure 1:**
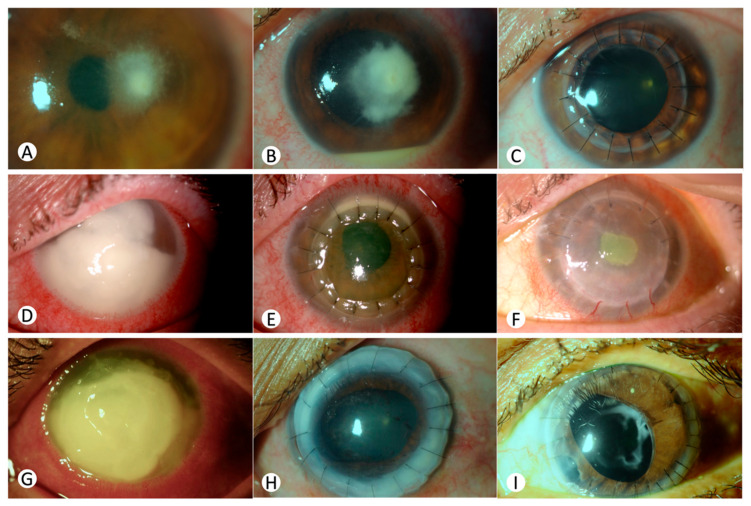
(**A**–**C**) **First case**. Clinical progression of corneal abscess caused by *Fusarium* sp. in a 51-year-old female: (**A**) initial presentation at our hospital after three months of corneal abscess, undergoing treatment with topical and systemic voriconazole; (**B**) evolution despite antifungal therapy; (**C**) status 24 h post-therapeutic TPK, demonstrating the absence of infectious manifestations with resolved anterior chamber activity. (**D**–**F**) **Second case.** Clinical progression of corneal abscess caused by *Fusarium* Solani in a 61-year-old female: (**D**) first visit at our hospital with one-month evolution corneal abscess and visual acuity of light perception; (**E**) status 24 h post-TPK, minor superior infiltration at the host interface; (**F**) four months post-transplantation, persistent central corneal epithelial defect, and stromal thickening but no active infection. (**G**–**I**) **Third case**. Clinical progression of corneal abscess caused by *Fusarium* spp. in a 56-year-old female: (**G**) rapidly evolving corneal ulcer exhibiting full-thickness corneal involvement and anterior chamber reaction; (**H**) status 24 h after a large-diameter TPK, with no residual infection; (**I**) status one month after the second large-diameter PK and Ahmed glaucoma valve implantation. *Due to an internal server error, further images of the third case were lost and could not be retrieved.*

A 51-year-old woman, a regular contact lens user with no significant medical history, presented to our center with a 3-month episode of *Fusarium* sp. keratitis. Despite topical and systemic voriconazole treatment (200 mg twice daily) alongside anti-inflammatory and antibiotic ophthalmic preparations, her visual acuity was reduced to 0.1 (decimal scale). Examination revealed a 3 × 3 mm corneal abscess without anterior chamber reaction (Figure 1A). Her condition worsened (Figure 1B), and the voriconazole therapy was supplemented with intrastromal injections. She was hospitalized for intensive antifungal treatment with hourly amphotericin B drops. Subsequent therapeutic keratoplasty was successful. Postoperatively, she showed no infection and improved anterior chamber activity, except for corneal synechiae (Figure 1C). Systemic voriconazole was discontinued a month post-surgery, and topical voriconazole and dexamethasone were tapered over three months. At six months, her best corrected visual acuity (BCVA) improved to 0.9.

### 3.2. Case 2 (Figure 1D–F)

A 61-year-old myopic woman, a contact lens user with no additional medical history, presented with a geographic corneal ulcer, initially treated as herpetic keratitis with valaciclovir and antibiotics. Subsequent examination revealed severe keratitis with extensive infiltration, a marked anterior chamber reaction, and a significant hypopyon, which led to a regimen of intensive topical antifungal and antibacterial therapy.

One month later, she presented to our center with vision reduced to light perception, exhibiting a large corneal abscess and hypopyon (Figure 1D) but no vitreous involvement on the B scan. Due to the treatment’s poor efficacy, she underwent an uncomplicated emergency therapeutic penetrating keratoplasty (TPK) with intracameral antifungals. Postoperatively, her regimen included systemic ciprofloxacin, topical dexamethasone, cycloplegics, vancomycin, ceftazidime, voriconazole, and natamycin.

The initial postoperative evaluation revealed minor superior infiltration at the host interface (Figure 1E). Upon identification of the *Fusarium* solani complex one week after TPK, the treatment was adjusted to focus on voriconazole and natamycin, which were progressively tapered over the subsequent two months. Four months after TPK, her BCVA was at the level of counting fingers, with persistent central corneal epithelial defects and stromal thickening but no active infection (Figure 1F). She was managed with daily moxifloxacin and dexamethasone. Consideration for a secondary keratoplasty is pending stabilization.

### 3.3. Case 3 (Figure 1G–I)

A 56-year-old woman with no medical history presented with a rapidly progressing corneal ulcer, refractory to topical antibiotics, and characterized by full-thickness involvement and hypopyon within a week. Suspecting a fungal origin, hourly amphotericin B and topical/systemic voriconazole treatment were initiated. Despite this, persistent deterioration (Figure 1G) and isolation of *Fusarium* sp. necessitated intracameral and intrastromal voriconazole, followed by large-diameter therapeutic keratoplasty. Although the surgery eradicated the infection (Figure 1H), she required further intervention, including corneal suturing and a conjunctival flap, for postoperative anterior chamber leakage. Systemic voriconazole was discontinued two months postoperatively, and the topical therapy was tapered over the following two months.

Subsequent complications included 360° anterior synechiae, leading to the implantation of an Ahmed glaucoma valve. Despite systemic immunosuppression with mycophenolate, another large-diameter PK was performed two years later (Figure 1I). Four years on, with her visual acuity limited to counting fingers, a third, smaller-diameter keratoplasty is being considered to restore vision.

### 3.4. Case 4 (Figure 2)

**Figure 2:**
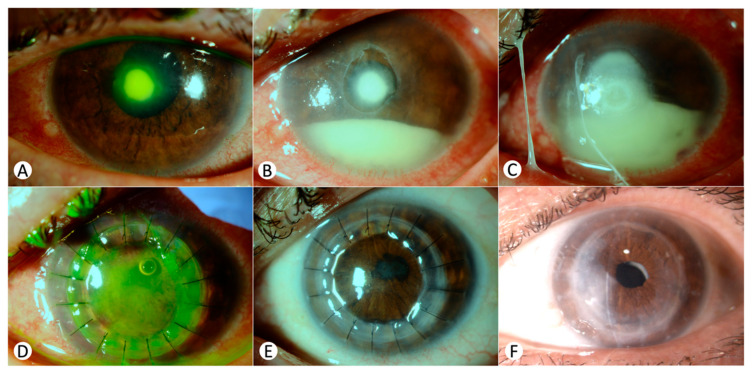
Clinical progression of corneal fungal abscess in a 60-year-old female: (**A**) initial diagnosis showing a central corneal abscess without anterior chamber inflammation; (**B**,**C**) important aggravation of the infection, with progressive hypopyon and iridian synechiae; (**D**) status 48 h post-TPK, demonstrating initial postoperative condition with lack of epithelialization of the graft; (**E**) twenty days post-transplantation, showing no recurrence of infection; (**F**) two years post-transplantation, after viscosynechialysis and cataract extraction with intraocular lens implantation surgery.

A 60-year-old woman with a background of daily contact lens use and past refractive surgery and no other medical history presented with a corneal abscess resistant to first-line antibiotics (Figure 2A), leading to hypopyon and iridial synechiae (Figure 2B,C). Suspected fungal infection led to treatment with topical voriconazole and amphotericin B, which did not prevent disease progression. Intrastromal and intracameral injections of tobramycin, voriconazole, and cefuroxime were initiated. Despite systemic voriconazole and repeated injections, her condition worsened. Cultures identified *Fusarium* sp., which showed resistance to amphotericin and partial resistance to voriconazole; topical natamycin was initiated without clinical improvement.

The therapeutic keratoplasty was performed one month after presentation due to the relentless disease (Figure 2D). Postoperatively, significant synechiae contributed to elevated intraocular pressure, which was managed with antihypertensive therapy. No signs of infection were noted during subsequent follow-up (Figure 2E). Systemic voriconazole was stopped after one month, and topical natamycin after four months. Subsequent viscosynechialysis and cataract surgery improved her BCVA to 0.4 (Figure 2F).

### 3.5. Case 5 (Figure 3)

**Figure 3:**
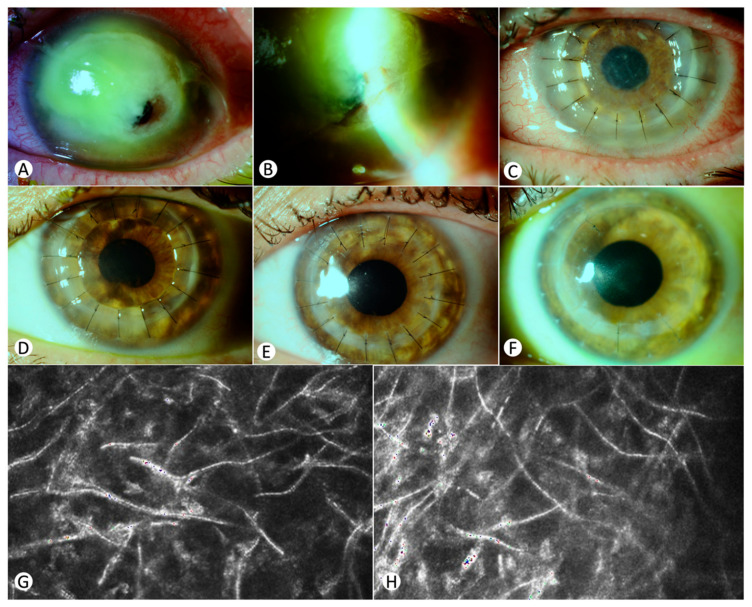
Clinical progression of corneal perforation in a 33-year-old female due to fungal abscess: (**A**,**B**) initial diagnosis showing a full-thickness corneal abscess with evident corneal perforation; (**C**) status 24 h post-therapeutic TPK, indicating initial postoperative condition; (**D**) twenty days post-transplantation, showing a residual temporal infectious abscess; (**E**) two months post-transplantation, with no residual infection and gradual reduction of vancomycin treatment; (**F**) three years post-keratoplasty, demonstrating sustained corneal transparency; (**G**,**H**) in vivo confocal microscopy (IVCM) at presentation. The procedure was performed with a Heidelberg Retina Tomograph with Rostock Cornea Module; (**G**) fungal hyphae at the epithelial depth (0 µm) varying in length, reflectivity, and width highly suggestive of *Fusarium* spp.; (**H**) fungal hyphae extending deeper into the stroma to 245 µm and 289 µm.

A 33-year-old woman with a history of nephrolithiasis and no significant ophthalmic history beyond contact lens use presented with corneal perforation and a BCVA limited to light perception (Figure 3A,B). She had been followed up with for a corneal ulcer for 40 days elsewhere, where she received topical antibiotics without improvement. In vivo confocal microscopy (IVCM) revealed filamentous structures suggestive of fungal infection, though initial cultures were negative (Figure 3G,H).

Emergency therapeutic keratoplasty was performed due to the perforation and strong suspicion of fungal etiology, excising most of the diseased tissue (Figure 3C). The postoperative regimen included topical voriconazole, natamycin, vancomycin, ceftazidime, chlorhexidine, and dexamethasone, in addition to systemic voriconazole. Histopathology showed fungal hyphae throughout the corneal thickness. A residual abscess at the temporal margin was noted (Figure 3D), but the graft remained stable with minimal edema and satisfactory epithelialization. Complications included posterior synechiae, managed with mydriatic agents. At the one-month follow-up, the examination revealed the resolution of infectious infiltrates. Antifungal therapy was gradually tapered and discontinued by the fifth month (Figure 3E). One year later, cataract extraction was conducted. Three years post-keratoplasty, her BCVA improved to 0.7 (Figure 3F).

**Table 1:** Summary of cases.

Case	Presentation	Post-KP Treatments	Results or Complications
Case 1: 51-year-old Woman	3-month episode of corneal ulcer.	Systemic: voriconazole. Topical (3 months): voriconazole and dexamethasone	Only some anterior synechiae BCVA: 0.9
Case 2: 61-year-old Woman	Contact lens user. Geographic ulcer misdiagnosed as herpetic keratitis evolved to extensive infiltration and hypopyon.	Systemic: ciprofloxacin. Topical (2 months): cycloplegics, vancomycin, ceftazidime, voriconazole, and natamycin.	Minor infiltration on the host interfaceBCVA: Counting fingersConsidering secondary keratoplasty upon stabilization
Case 3: 56-year-old Woman	Fast evolving corneal ulcer to full-thickness involvement and hypopyon in one week.	Systemic: voriconazole. Topical (2 months): voriconazole and dexamethasone.	Postoperative Seidel requiring additional suturing and a conjunctival flap. Developed 360° anterior synechiae leading to Ahmed valve implantation. Required secondary keratoplasty after 2 years. At 4 years with BCVA of counting fingers a third smaller-diameter keratoplasty is under consideration.
Case 4: 60-year-old Woman	Contact lens user. Fast-evolving corneal abscess resistant to antibiotics leading to hypopyon and iridial synechiae	Systemic: voriconazole Topical (4 months): natamycin and corticosteroids.	Intraocular hypertension due to posterior synechiae managed with topical antihypertensive therapy. Subsequent viscosynechiolysis and cataract surgery improved BCVA to 0.4
Case 5: 33-year-old Woman	Followed for 40 days for a corneal ulcer. Came to our center with a corneal perforation.	Systemic: voriconazole Topical (5 months): voriconazole, natamycin, vancomycin, ceftazidime, chlorhexidine, and dexamethasone.	Residual infiltration on host cornea. Posterior synechiae managed with mydriatic agents.BCVA 0.7

## 4. Discussion

Fungal keratitis emerges as a significant concern for ocular health, with *Fusarium* spp. accounting for 61% of cases, followed by Aspergillus and Candida.^2^ These fungi are prevalent in soil and water, especially in lower-income tropical and subtropical areas [1,7], yet cases in developed countries are on the rise, often associated with contact lens use [1,2].

Commonly misdiagnosed, fungal keratitis is sometimes initially treated with antibiotics. The first culture growth is typically observed at 72 h for 82% of cases, and by seven days in 97% [7]; these periods contribute significantly to the delay in initiating effective antifungal care [1]. Although molecular diagnostics could expedite identification, Sabouraud agar remains a gold standard [1].

In vivo confocal microscopy (IVCM) facilitates early detection of keratomycosis by revealing fungal filaments (Case 5, Figure 3G,H) and could serve as a prognostic tool, with filament count correlating with disease severity [2]. Direct microscopic examination of stained samples is also invaluable for detecting fungi, aiding swift and accurate diagnosis and management [1,7]. Given that late diagnosis is one of the main reasons some cases of *Fusarium* keratitis necessitate TKP, it is crucial to employ, if possible, molecular diagnostic methods such as PCR, and in vivo confocal microscopy (IVCM) [2]. These techniques can provide much faster diagnoses, although cultures remain the gold standard [1].

The treatment of *Fusarium* keratitis encompasses topical antifungals, which may be augmented with subconjunctival or intrastromal injections, alongside oral antimycotic agents [7]. The fungal resistance to most antifungal drugs poses a significant treatment challenge [3]. Topical 1% voriconazole and 5% natamycin are currently among the most effective available treatments [1,7], with the amphotericin B lipid complex serving as an alternative [7]. Oral voriconazole or ketoconazole are additional options. The resistance to antifungal drugs makes these cases stand out due to their potential to cause perforation, as seen in the last case. Being able to perform this surgery is critical once reaching these rare but very dangerous stages. Corneal cross-linking has potential as an adjunctive treatment for reducing corneal hyphae through oxidation; however, it is only indicated in the early stages of the disease [7]. When these approaches fail, therapeutic keratoplasty is performed to excise the infection and preserve or restore the eye’s integrity [7]. The first four cases underwent therapeutic keratoplasty due to the absence of or poor response to topical or systemic treatment, while the last patient had a corneal perforation, and therapeutic keratoplasty was performed as an emergency. The excised cornea was analyzed both microbiologically and histopathologically to identify the pathogen and assess the infection’s extent [7].

This technique can save the patient’s eye; however, postoperative complications such as infection recurrence, graft rejection, or secondary glaucoma remain significant concerns [1]. Despite the absence of a universal consensus on the timing of surgery, it is widely acknowledged as a necessary intervention for *Fusarium* spp. fungal keratitis, particularly when clinical conditions fail to improve or deteriorate [8]. Prompt transplantation is recommended to remove the infection and maintain the eye’s function and structure. For superficial infections, deep anterior lamellar keratoplasty (DALK) can be an alternative, but it risks incomplete removal of fungal hyphae. Due to *Fusarium*’s tendency for full-thickness corneal invasion [1], DALK should be limited to select cases without deep ulcers [9,10]. The primary objective of a therapeutic penetrating keratoplasty (TPK) is to eliminate the infection; therefore, the outcome of this procedure should be assessed on these terms, with gains in visual acuity considered a secondary priority. In some cases, as demonstrated in our practice, a secondary penetrating keratoplasty may be required. When performing a TPK, the criteria for selecting donor tissue are less stringent, and given that the recipient’s eye is often in a compromised state, restoration of visual acuity may not occur as rapidly as it might with a regular keratoplasty.

This manuscript has several limitations. The small sample size from a single center restricts the generalizability of the results. Furthermore, patient presentations vary significantly, as some were referred from other hospitals, resulting in diverse initial conditions. Additionally, the outcomes of TPK depend on the quality of the donor tissue, the overall condition of the patient at the time of surgery, and the systemic treatments.

In conclusion, the increasing incidence of fungal keratitis underscores the need for corneal specialists to be proficient in therapeutic keratoplasty (TPK). This vital surgical technique complements medical therapy for the effective management of *Fusarium* keratitis. While this manuscript provides practical examples and lays some groundwork for deciding when to perform a TPK, precise guidelines and protocols are still lacking for making universal decisions. Further studies are essential to establish these guidelines, which would help standardize care and improve outcomes in the management of fungal keratitis.

## Data Availability

The original contributions presented in this study are included in the article. Further inquiries can be directed to the corresponding author.
